# Serotonin and Dopamine Show Different Response Profiles to Acute Stress in the Nucleus Accumbens and Medial Prefrontal Cortex of Rats with Neuropathic Pain

**DOI:** 10.1007/s11064-023-03906-y

**Published:** 2023-03-20

**Authors:** James W. M. Kang, Kevin A. Keay, Michael D. Kendig, Laura H. Corbit, David Mor

**Affiliations:** 1 School of Medical Sciences [Neuroscience], The University of Sydney, Sydney, NSW 2006 Australia; 2 School of Life Sciences, University of Technology Sydney, Sydney, NSW 2007 Australia; 3 Department of Psychology, The University of Toronto, Toronto, ON M5S 3G3 Canada

**Keywords:** Pain, Stress, Nerve-injury, Dopamine, Serotonin, Medial prefrontal cortex

## Abstract

The ability to adaptively guide behaviour requires the integration of external information with internal motivational factors. Decision-making capabilities can be impaired by acute stress and is often exacerbated by chronic pain. Chronic neuropathic pain patients often present with cognitive dysfunction, including impaired decision-making. The mechanisms underlying these changes are not well understood but may include altered monoaminergic transmission in the brain. In this study we investigated the relationships between dopamine, serotonin, and their metabolites in key brain regions that regulate motivated behaviour and decision-making. The neurochemical profiles of the medial prefrontal cortex, orbital prefrontal cortex, and nucleus accumbens were analysed using HPLC in rats that received a chronic constriction injury (CCI) of the right sciatic nerve and an acute stress (15-min restraint), prior to an outcome devaluation task. CCI alone significantly decreased dopamine but not serotonin concentrations in the medial prefrontal cortex. By contrast, restraint stress acutely increased dopamine in the medial prefrontal cortex, and the nucleus accumbens; and increased serotonin in the medial prefrontal cortex 2 h later. The sustained dopaminergic and serotonergic responses to acute stress highlight the importance of an animal’s ability to mount an effective coping response. In addition, these data suggest that the impact of nerve injury and acute stress on outcome-devaluation occurs independently of dopaminergic and serotonergic transmission in the medial prefrontal cortex, orbital prefrontal cortex and nucleus accumbens of rats.

## Introduction

Selecting appropriate behaviours is essential for successfully navigating the surrounding environment, and external factors that undermine this ability, such as stress, can reduce an individual’s quality of life [1]. Decision-making can be considered as a set of biological and psychological processes that guide and direct behaviour towards a specific goal or outcome. This requires the organism to integrate internal motivating factors (hunger, thirst) with acquired knowledge of the relationship between an action and its outcome [2]. Goal-directed behaviour’s are sensitive to changes in outcome value, such that the decision to perform or withhold an action is influenced by the expected outcome. Animal models used to investigate decision-making processes often employ an outcome devaluation test to observe whether the behaviour is under goal-directed control [3].

Neuropathic pain frequently presents with comorbid stress related conditions such as anxiety, depression and sleep disturbances, all of which commonly presents with cognitive dysfunction such as impairments in decision-making [4, 5]. Cognitive impairments are more likely to occur in the neuropathic pain state in the presence of stress-related conditions such as anxiety [6]. Further, alterations in an individual’s behaviour are often exacerbated under situations of stress, and are often interpreted as an impairment of motivational drive, emotional regulation and/or decision-making processes [7]. Our previous studies have shown that rats with a chronic constriction injury (CCI) of the right sciatic nerve, show reduced sensitivity to outcome devaluation after an acute restraint stress [8]. Further, we have shown that CCI results in lateralised changes in dopamine (DA) and serotonin (5-HT) levels in the dorsal striatum, a region critical for learning, goal-directed behaviours and pain processing and thus these changes have been implicated in the nerve-injury dependent regulation of behavioural control processes [9].

The dopaminergic system has been heavily implicated in the control of habitual and goal-directed motivational behaviours [10]. For example, exposure to amphetamines, which enhances dopamine transmission, promotes the transition from goal-directed behaviours to habitual behaviours [11, 12], and is thought to play a critical role in habit formation [13, 14]. Goal-directed motivational behaviours also appears to require dopaminergic transmission via circuitry including the medial prefrontal cortex and nucleus accumbens [15–17]. Further, the serotonergic system is implicated in behavioural selection and inhibition [18]. In the clinical setting, patients with impulsive disorders including aggression, violence and mania show reductions in serotonin metabolites measured in the cerebral spinal fluid [19, 20]. In addition, the depletion of tryptophan, an amino acid crucial for serotonin synthesis, promotes habitual behaviours over goal-directed behaviours during appetitive tasks in healthy humans [21]. This has led to the suggestion that dopamine and serotonin act together to coordinate behavioural selection [22]. It has been shown consistently that alterations in dopaminergic and serotonergic neurotransmission accompany persistent pain states [23–25]. However, the regional specificity of changes in these systems immediately after a decision-making task is not well understood.

To expand our previous work in the dorsal striatum, here we investigate the relationship between dopamine (DA) and serotonin (5-HT) in key brain regions involved in directing motivated behaviours and decision-making processes. In two separate experiments, we studied whether alterations in DA and 5-HT neurochemistry in the medial prefrontal cortex (mPFC), orbital prefrontal cortex (oPFC) and nucleus accumbens (NAc) are related to impairments in goal-directed behaviours in neuropathic rats. In Experiment 1 these relationships were quantified in rats with a nerve injury (INJURED); and in Experiment 2 we studied the additional impact of an acute stressor in nerve-injured rats (INJURED + STRESS). In both experiments, data were compared to both sham-injured and un-injured, naïve control groups.

## Materials and Methods

### Animals

All experimental procedures were carried out with the approval of the Animal Care and Ethics Committee at the University of Sydney (AEC No. 2014/561) and in accordance with the guidelines of the Code for the Care and Use of Animals in Research Australia, and the Ethical Guidelines for Investigation Association for the Study of Pain [26]. The present study used 84 brains from adult male Sprague–Dawley rats that underwent outcome-devaluation testing and CCI of the right sciatic nerve. The behavioural data from these rats were reported previously in Mor et al. [8] and Gemikonakli et al. [9], and dorsal striatum neurochemical data were presented in Gemikonakli et al. [9].

### Experimental Design

Adult Male Sprague–Dawley rats (200–260 g on arrival) were sourced from the Animal Resource Centre (Perth, WA, Australia). Rats were group housed (*n* = 4/cage) in large, individually ventilated cages with wood shaving as bedding and regulated airflow (temperature 22 ± 3 °C, humidity 70% ± 20%) on a reverse dark–light cycle (12:12, lights off 09:00–21:00). Rats received ad libitum access to tap water and standard laboratory chow during the habituation period. Surgical procedures and behavioural testing were performed during the dark cycle.

Rats were acclimated for 7 days after arrival and were then arbitrarily allocated to one of three groups: chronic constriction injury (*CCI*) (*n* = 29), *Sham-injury* (*n* = 26) which underwent surgical procedure without nerve ligation or un-injured controls, *Naïve* (*n* = 29), which were left in their home-cage on the day of surgery.

#### Surgical Procedures

Rats were anaesthetised by induction with isoflurane (5% in 100% oxygen), delivered via an airtight induction chamber. Surgical level anaesthesia was maintained with 2–3% isoflurane in 100% oxygen administered via a custom-made facemask for approximately 20 min. Body temperature was maintained at 37 ± 1 °C using a homeostatically controlled heating blanket and rectal thermometer. CCI was performed as described by Bennett and Xie [27], the right sciatic nerve was exposed via blunt dissection through the bicep femoris muscle, and four chromic gut ligatures (chromic gut, 5–0 Ethicon, Johnson & Johnson Medical) were tied loosely 1 mm apart, just proximal to the trifurcation of the sciatic nerve. The nerve was then repositioned to its original location and the skin was closed by the application of four to six Michel clips and a topical antibiotic powder mixed with Vaseline was applied as a paste onto the closed incision site. Sham surgical procedures included blunt dissection and exposure of the right sciatic nerve, but no ligatures were applied. On the day after surgery (day 8), all groups were allowed to recover for 24 h before the start of food restriction.

#### Behavioural Training and Testing

**Fig. 1:**
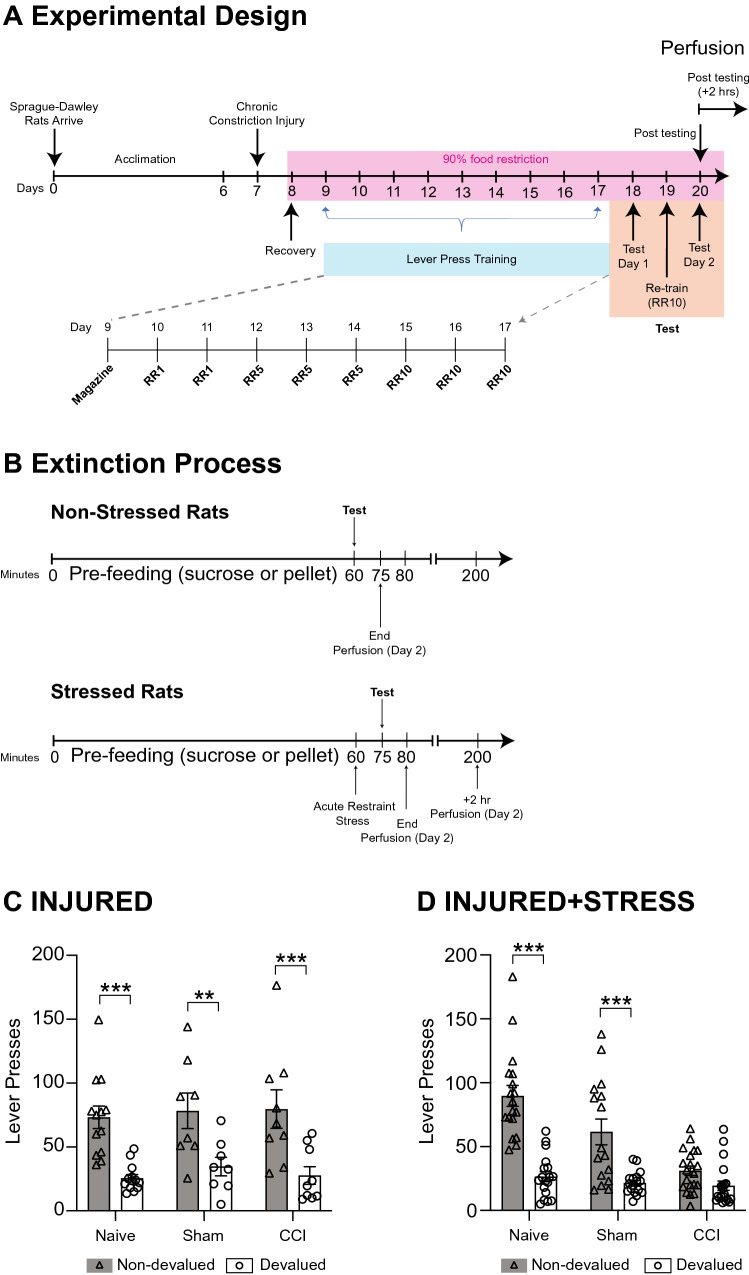
Experimental timeline. **A** Adult Sprague–Dawley rats were acclimatised to home cages for 7 days (Days 0–6). On day 7 rats received a chronic constriction injury (CCI, *n* = 29), sham-injury (Sham, *n* = 26) or non-procedure (Naïve, *n* = 29). Day 8 all rats were allowed to recover. From day 8 food access was placed on a restricted feeding schedule of 90% of free-feeding weight to motivate rats to complete instrumental training. On day 9, a single session of magazine training was given. On days 10 and 11 a continuous reinforcement schedule was utilised using a random-ratio (RR) schedule of 1. Days 12–14 a RR5 was used, and a RR10 from days 15–17. Two devaluation tests were performed on days 18 and 20 which involved an extinction process. On day 19 re-training using a RR10 was performed. **B** Outcome devaluation involved pre-feeding rats with either pellets (15 g) or sucrose solution ((80 ml) 20% (w/v)) for 1-h in individual pre-feeding cages. Non-stressed rats were immediately placed into operant chambers for a 5-min extinction test where the levers were present, but no rewards delivered and were euthanised immediately after the test. Stressed rats received a 15-min acute restraint stress then were immediately placed into the operant chambers for a 5-min extinction test. Stressed rats were euthanised immediately after testing and Stressed (+ 2 h) rats were euthanised 2 h after testing. **C** The sensitivity to outcome-devaluation test is shown for rats that received a CCI, but no acute restraint stress (INJURED). **D** Sensitivity to outcome-devaluation test is shown for rats that received both a CCI and an acute restraint stress (INJURED + STRESS). **P < 0.01, ***P < 0.001 graphs presented in this figure (**C, D**) are selected data which have been re-graphed from (Mor et al. [8]; Gemikonakli et al. [9])

Instrumental training and outcome-devaluation testing procedures were performed as described in detail by Mor et al. [8]. In brief, following recovery from surgery (day 8), rats were food restricted (15 g chow/rat/day) to motivate performance in instrumental training. Body weight and physical condition of the rats were monitored daily. Instrumental training (days 9–17) was conducted in 12 operant chambers (Med-Associates, USA) with two retractable levers located on each side of the chamber and a recessed magazine centred between them. Rats were trained to press two levers to earn either 15 pellets or 15 sucrose (0.1 ml, 20% w/v) rewards in daily sessions as outlined in Fig. 1A. Rats initially received a single session of magazine training (day 9), in which 15 pellets or 15 sucrose (0.1 ml, 20% w/v) rewards were delivered to the magazine on a random-time 60 s schedule. For instrumental training (days 10–17), the left and right levers were assigned to earn either pellets or sucrose in a counter-balanced fashion within each group (i.e., left = sucrose, right = pellet OR, right = pellet, left = sucrose). Training consisted of two sessions a day, with one session per lever (and reward) with a minimum 60-min break between each training session per rat. Each training session was completed when 30 outcomes were earned or after 45 min had elapsed. The order of training sessions (pellet or sucrose) was alternated each day.

The ability of the rat to perform goal-directed behaviour was tested using outcome devaluation by sensory-specific satiety on days 18 and 20, with a re-training day on day 19. Outcomes were devalued by sensory-specific satiety in which rats were allowed to freely consume one of the two outcomes for 1 h prior to testing (see Fig. 1A-B).

Following consumption, rats in Experiment 1 (INJURED—*CCI* *N* = 9; *Naïve N* = 12, *Sham-injured N* = 8) were placed into the operant chambers immediately after pre-feeding with both levers inserted for a 5-min extinction test (no outcomes were delivered). Magazine entries and lever presses were recorded. Rats in Experiment 2 were assessed either immediately after acute restraint stress, or 2 h later (INJURED + STRESS—*CCI* + *stress N* = *10; CCI* + *stress[2 h] N* = *10; Naïve* + *stress N* = *9; Naive* + *stress[2 h] N* = *8; Sham* + *stress N* = *9; and Sham* + *stress[2 h] N* = *9*). Stressed rats were restrained for 15-min using a canvas sleeve (“Snuggle”, Lomir Biomedical Inc) immediately after pre-feeding in a separate test room, and then were placed into the operant chambers for a 5-min extinction test identical to INJURED groups. Sensitivity to outcome-devaluation by sensory-specific satiety has been observed up to 2 h after pre-feeding and as such we do not believe the 15 min delay in testing between stressed and non-stressed rats undermined the manipulation [28]. Our previously published behavioural data also show no significant differences between Naïve and Naïve + stress rats in sensitivity to outcome-devaluation (see Fig. 1C and D). Figure 1C and D show the sensitivity to outcome-devaluation was not affected in INJURED experimental groups. Further, INJURED + STRESS showed reduced sensitivity in outcome-devaluation in rats that received a CCI. Behavioural data were previously published in [8] and Gemikonakli, et al. 2017.

In this study, we sought to investigate the neurochemical profiles of prefrontal cortical and accumbal regions of rats with nerve injury following acute stress. INJURED (Experiment 1) and INJURED + STRESS (Experiment 2) experimental groups were compared either immediately after, or 2 h after, an outcome-devaluation test.

#### Perfusion

As shown in Fig. 1, immediately after the second devaluation test rats from Experiment 1 (*CCI* = 9; *Naïve* = 12, *Sham-injured* = 8) were deeply anaesthetised (sodium pentobarbitone, 120 mg/kg, i.p.) and perfused intra-cardially with ice cold saline immediately following the second devaluation test (see Fig. 1A, B). The brains were removed and immediately snap frozen in liquid nitrogen and stored at −80 °C until micro-dissection.

Rats from Experiment 2 (INJURED + STRESS) underwent identical perfusion and brain extraction procedures either: (i) immediately after the second devaluation test (*CCI* + *stress N* = *10; Naïve* + *stress N* = *9; Sham* + *stress N* = *9*); or (ii) 2 h after (*CCI* + *stress[2 h] N* = *10; Naive* + *stress[2 h] N* = *8; Sham* + *stress[2 h] N* = *9*) the second devaluation test (see Fig. 1A, B). The additional tissue collection 2 h after devaluation testing was selected to provide further information on the time-course of neurotransmitter clearance.

#### Brain Dissection

##### Medial Prefrontal Cortex (mPFC)

All dissections were performed under a binocular microscope. The medial prefrontal cortex was first isolated by sectioning the brain in a coronal plane at: (i) the point at which the olfactory bulbs meet the rostral pole of the frontal cortices (rostral boundary) and, (ii) 1 mm anterior to the closure of the rhinal fissure (caudal boundary). The medial prefrontal cortex (*infralimbic, prelimbic* and *anterior cingulate regions*) was subsequently isolated by a parasagittal cut at the medial border of the forceps minor of the corpus callosum.

##### Orbital Prefrontal Cortex (oPFC)

The orbital prefrontal cortex (*ventrolateral orbital, lateral orbital* and *anterior insular regions*) was isolated by a horizontal cut below forceps minor of the corpus callosum. The remainder of the frontal block (anterior to the optic chiasm) was micro-dissected to isolate the nucleus accumbens (NAc) as described below.

##### Nucleus Accumbens (NAc)

In brief, a coronal cut was made anteriorly at the rhinal fissure (+ 2.5 mm to bregma) and posteriorly at the optic chiasm (0.0 mm to bregma). The block was then cut horizontally at the floor of the lateral ventricle, the dorsal striatum was separated from the NAc in the ventral striatum. The left and right NAc were isolated from the surrounding septal structures.

### Neurochemical Analysis: High Performance Liquid Chromatography

Dissected tissue was homogenised by sonication (30% duty cycle, output 3) in 19 × volume (per wet weight in mg of brain tissue) homogenisation buffer (150 mM phosphoric acid, 500 μM diethyene triamine penta-acetic acid in MilliQ purified water). Following homogenisation, the brain tissue was centrifuged for 25 min at 16,000 rpm at 4 °C to separate the cell debris from the supernatant containing the total protein. The supernatant was removed, 2 μl was taken for total protein quantification using a *direct detect*© Spectrophotometer (Millipore) and the remainder was passed through a 3 kDa Amicon Ultra Centrifuge filter (Millipore) at 16,000 rpm in a centrifuge at 4 °C for 90 min to remove large proteins from the sample for subsequent high performance liquid chromatography (HPLC) analysis.

The HPLC systems consisted of a Phenomenex Gemini C18 column (5 μm: 150 × 4.6 mm) maintained at 25 °C, and a pressure pump (Shimadzu Prominence LC-240AD, Shimadzu, Kyoto, Japan) which circulated the mobile phase (13% methanol, 87% 0.01 M monobasic sodium phosphate, 0.1 mM ethylenediaminetetraacetic acid (EDTA), 0.65 mM 1-octane sulfonic acid, 0.5 mM triethylamine at pH 2.81) at a flow rate of 1.5 ml/min. Sample injections were performed by a Shimadzu Prominence SIL-20A auto-sampler. The electrochemical detection established the retention time of the injected substance using a glassy carbon electrode set to a positive potential of 0.85 V, relative to an Ag/AgCl reference electrode. The signal from the detector was recorded and analysed using a Shimadzu CLASS-VP lab solutions version 6.11 data acquisition software. External standards were run daily for each neurotransmitter or metabolite to produce a six-point calibration curve.

### Statistical Analysis

Statistical analyses were performed in R version 4.1.0 (R Core Team, 2021) using the RStudio version 1.4.1717 (RStudio Team) using the following packages: *emmeans* (v.1.4.4; Length [29], *tidyverse* (v1.2.1; Wickham et al. [30] and *rstati*× (v0.7.0; Kassambara [31].

Differences between the concentrations of the neurotransmitters dopamine (DA) and serotonin (5-HT) and their metabolites 3, 4-Dihydroxyphenylacetic acid (DOPAC), Homovanillic acid (HVA), 5-Hydroxyindoleacetic acid (5HIAA), were assessed in a three-way ANOVA (*stress × surgery × side*) in the mPFC, oPFC and NAc. This allowed for differences between brain hemispheres of the same rat (within-subjects factor, *side*) to be assessed alongside the impact of CCI and stress. Significant interaction effects were followed by pairwise comparisons with a Bonferroni adjustment to control for type-1 error rate. Data representation in figures are expressed as bar graphs with individual data points identified, error bars represent ± standard error of the mean (SEM).

## Results

Previously it was shown that the combination of nerve-injury and acute stress reduced the sensitivity to outcome-devaluation (see more [8], summarized in Fig. 1C and D).

### Experiment 1: INJURED

#### Dopaminergic Transmission

##### Medial Prefrontal Cortex

**Fig. 2:**
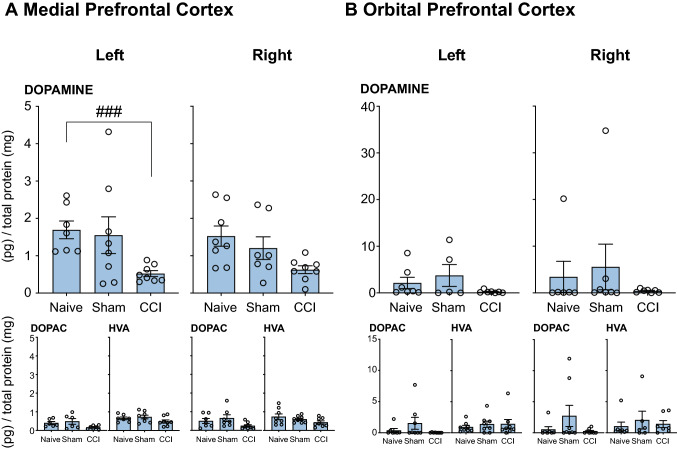
Neurochemical analysis of dopaminergic transmission in the **A** Medial Prefrontal Cortex and **B** Orbital Prefrontal Cortex. The dopamine, DOPAC and HVA concentration in the left and right mPFC and oPFC of rats that underwent of rats that underwent CCI with no acute restraint stress (INJURED—*Naïve, Sham, CCI*) are presented. Significant differences are represented by ^###^*P* < 0.001 compared to *Naïve* (surgery) groups. Three-way ANOVA, with simple effects pairwise comparisons with Bonferroni correction. Error Bars represent ± SEM

(i) *Dopamine:* Fig. 2 shows the effect of INJURED experimental groups (*Naïve*, *Sham-injured* or *CCI*) on the concentrations of dopamine and its metabolites DOPAC and HVA in the mPFC. Table 1 summarizes the significant main effects and interaction effects. Pairwise comparisons with a Bonferroni correction revealed that CCI significantly reduced dopamine concentrations in the left mPFC (contralateral to injury) when compared to *Naïve* rats (*P* < 0.001, Fig. 2A). The right mPFC showed a similar trend but did not reach statistical significance (Fig. 2A).
(ii) *DOPAC:* No changes were detected for DOPAC in the mPFC (Fig. 2A).
(iii) *HVA:* No changes were detected for HVA in the mPFC (Fig. 2A).

**Table 1:** Effects of CCI with or without acute stress on DA and 5-HT in the medial prefrontal cortex

	Dopamine	DOPAC	HVA	5-HT	5HIAA
Condition	*P*-value	*P*-value	*P*-value	*P*-value	*P*-value
Stress	***	***	***	***	ns
Surgery	***	ns	ns	ns	ns
Side (L v R)	**	ns	**	**	**
Stress × Surgery	ns	ns	ns	ns	ns
Stress × Side	**	*	***	ns	ns
Surgery × Side (L v R)	ns	ns	ns	ns	ns
Stress × Surgery × Side (L v R)	ns	ns	ns	ns	ns

*P < 0.05, **P < 0.01, ***P < 0.001 represent significant main or interaction effects following analysis with a three-way ANOVA

##### Orbital Prefrontal Cortex

*Dopamine/DOPAC/HVA:* No changes in dopamine, DOPAC or HVA were found in the oPFC (see Table 2 and Fig. 2B).

**Table 2:** Effects of CCI with or without acute stress on DA and 5-HT in the orbital prefrontal cortex

	Dopamine	DOPAC	HVA	5-HT	5HIAA
Condition	*P*-value	*P*-value	*P*-value	*P*-value	*P*-value
Stress	ns	ns	*	***	ns
Surgery	ns	ns	ns	ns	ns
Side (L v R)	ns	ns	ns	ns	***
Stress × Surgery	ns	ns	ns	ns	ns
Stress × Side (L v R)	ns	***	ns	ns	**
Surgery × Side (L v R)	ns	ns	ns	ns	ns
Stress × Surgery × Side (L v R)	ns	ns	ns	ns	ns

*P < 0.05, **P < 0.01, ***P < 0.001 represent significant main or interaction effects following analysis with a three-way ANOVA

##### Nucleus Accumbens

**Fig. 3:**
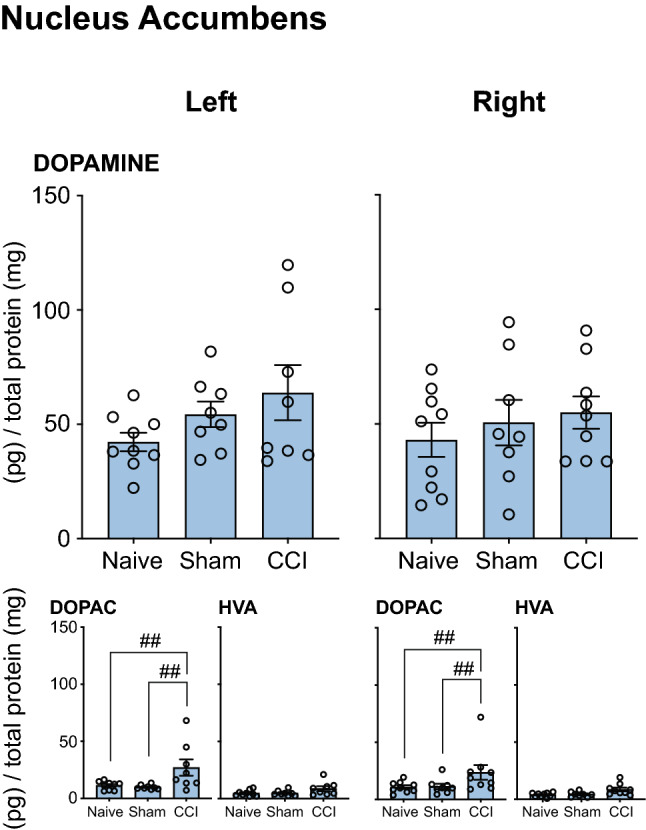
Neurochemical analysis of dopaminergic transmission in the Nucleus Accumbens. The dopamine (**A**), DOPAC (**B**) and HVA (**C**) concentration in the left and right NAc of rats that underwent CCI with no acute restraint stress (INJURED—*Naïve, Sham, CCI*) are presented. Significant differences are represented by ^##^*P* < 0.01 compared to *Naïve or Sham* (surgery) groups. Three-way ANOVA, with simple effects pairwise comparisons with Bonferroni correction. Error Bars represent ± SEM

*Dopamine/DOPAC/HVA*: No significant changes in dopamine or HVA were found in the NAc (Fig. 3A). There was a significant effect of CCI on DOPAC concentrations bilaterally in the NAc when compared to *Naïve* and *Sham-injured* rats (*P* < 0.01, Fig. 3). A summary of statistical results can be found in Table 3.

**Table 3:** Effects of CCI with or without acute stress on DA and 5-HT in the nucleus accumbens

	Dopamine	DOPAC	HVA	5-HT	5HIAA
Conditions	*P*-value	*P*-value	*P*-value	*P*-value	*P*-value
Stress	***	*	***	**	***
Surgery	ns	*	ns	ns	*
Side (L v R)	ns	*	ns	*	ns
Stress × Surgery	ns	*	ns	**	ns
Stress × Side (L v R)	**	***	ns	ns	ns
Surgery × Side (L v R)	ns	ns	ns	ns	ns
Stress × Surgery × Side (L v R)	ns	*	*	ns	ns

*P < 0.05, **P < 0.01, ***P < 0.001 represent significant main or interaction effects following analysis with a three-way ANOVA

#### Serotonergic Transmission

**Fig. 4:**
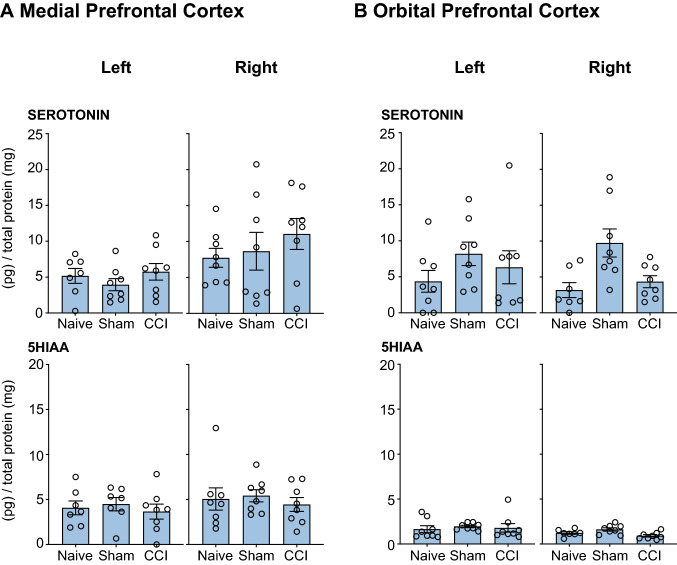
Neurochemical analysis of serotonergic transmission in the Medial Prefrontal Cortex and Orbital Prefrontal Cortex. The serotonin and 5HIAA concentration in the left and right mPFC and oPFC of rats that underwent CCI, but no acute restraint stress (INJURED—*Naïve, Sham, CCI*) is presented. Three-way ANOVA, with simple effects pairwise comparisons with Bonferroni correction. Error Bars represent ± SEM

**Fig. 5:**
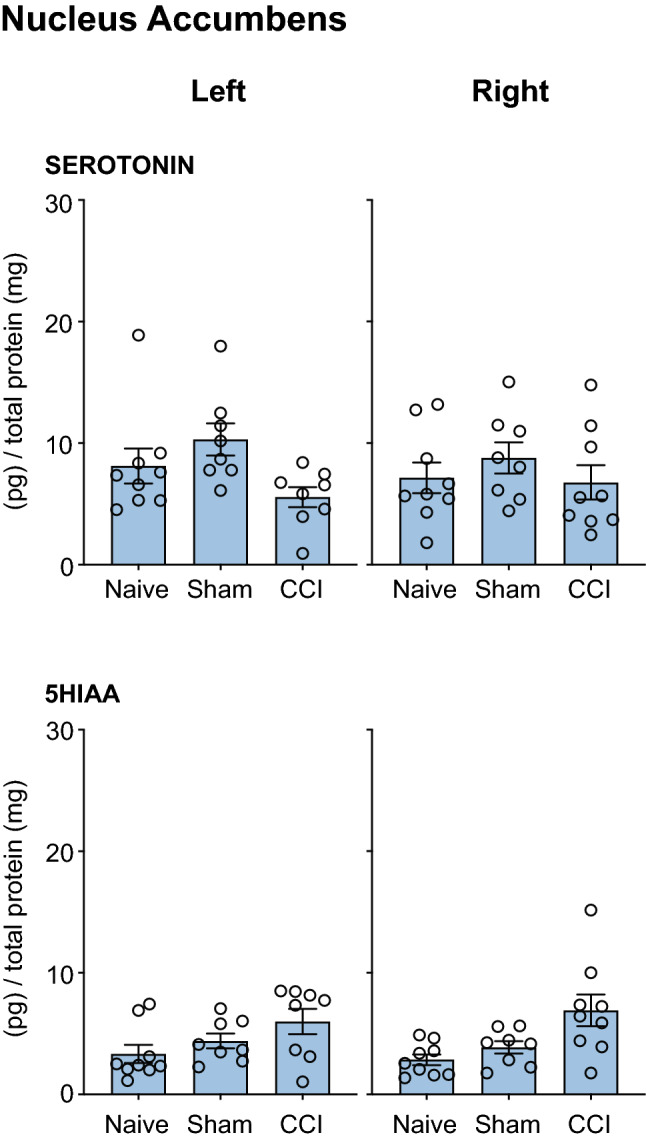
Neurochemical analysis of serotonergic transmission in the Nucleus Accumbens (NAc). The serotonin and 5HIAA concentration in the left and right NAc of rats that underwent CCI, but no acute restraint stress (INJURED—*Naïve, Sham, CCI*) are presented. Three-way ANOVA, with simple effects pairwise comparisons with Bonferroni correction. Error Bars represent ± SEM

There were no differences in the levels of serotonin or 5HIAA in *Naïve, Sham* and *CCI* groups in the medial prefrontal cortex (Fig. 4A); the orbital prefrontal cortex (Fig. 4B) or the nucleus accumbens (Fig. 5).

### Experiment 2: INJURED + STRESS

#### Dopaminergic Transmission

##### Medial Prefrontal Cortex

**Fig. 6:**
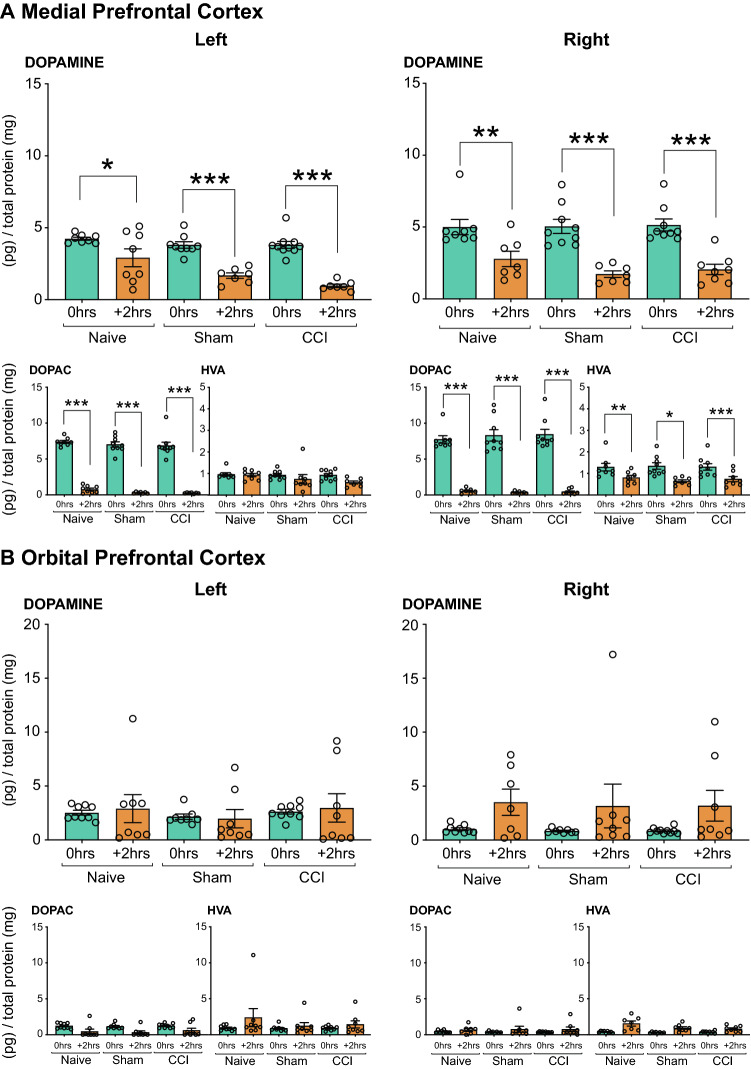
Neurochemical analysis of dopaminergic transmission in the **A** Medial Prefrontal Cortex and **B** Orbital Prefrontal Cortex. The dopamine, DOPAC and HVA concentration in the left and right of the mPFC and oPFC of rats that underwent CCI and acute restraint stress (INJURED + STRESS—*Naïve, Sham, CCI*) are presented. Significant differences are represented by *P < 0.05, **P < 0.01 and ***P < 0.001 when compared + *2 h* are compared to *0 h* groups*.* Three-way ANOVA, with simple effects pairwise comparisons with Bonferroni correction. Error Bars represent ± SEM

(i) *Dopamine*: There were no differences in dopamine levels in acutely stressed *Naïve, Sham* or *CCI injured* rats immediately after outcome devaluation. Similarly, there were no differences in dopamine levels in acutely stressed *Naïve, Sham* or *CCI injured* rats 2 h after outcome devaluation. There were however significant differences in dopamine concentrations between *Naïve, Sham* and *CCI injured* groups when the levels immediately after the outcome devaluation were compared to those 2 h later (*p* < 0.001) (see Fig. 6A).
(ii) *DOPAC:* There were no differences in DOPAC levels in acutely stressed *Naïve, Sham* or *CCI injured* rats immediately after outcome devaluation. Similarly, there were no differences in DOPAC levels in acutely stressed *Naïve, Sham* or *CCI injured* rats 2 h after outcome devaluation. The levels of DOPAC were significantly reduced 2 h after outcome-devaluation testing in all groups (*P* < 0.001) (Fig. 6A).
(iii) *HVA:* A Three-Way Mixed ANOVA for HVA revealed statistically significant interaction effects of Stress × Side (F_2, 59_ = 3.342, *P* = 0.043) and significant main effects of stress (F_2, 59_ = 31.735, *P* < 0.001). There were significant and lateralized decreases in HVA in the right mPFC of all groups 2 h after outcome-devaluation testing when compared to samples collected immediately after outcome-devaluation testing (see Fig. 6A).

##### Orbital Prefrontal Cortex

*Dopamine/DOPAC/HVA*: No changes in dopamine, DOPAC or HVA were found in the oPFC (see Table 2 and Fig. 6B).

##### Nucleus Accumbens

**Fig. 7:**
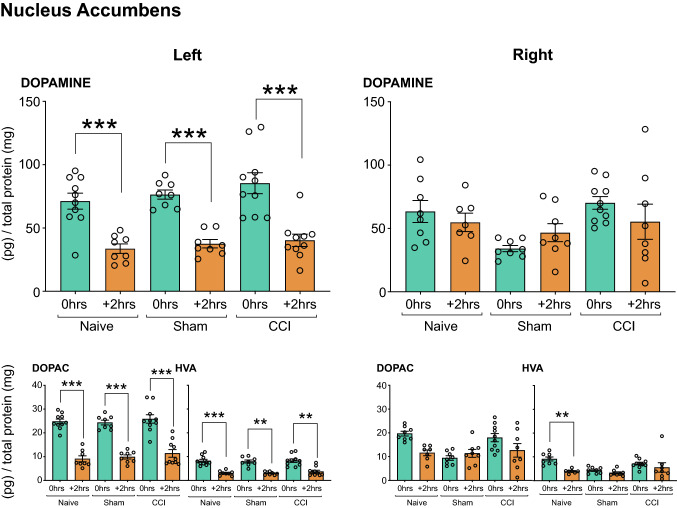
Neurochemical analysis of dopaminergic transmission in the Nucleus Accumbens. The dopamine, DOPAC and HVA concentration in the left and right NAc of rats that underwent CCI and acute restraint stress (INJURED + STRESS—*Naïve, Sham, CCI*) are presented. Significant differences are represented by **P < 0.01 and ***P < 0.001 when compared + *2 h* are compared to *0 h* groups*.* Three-way ANOVA, with simple effects pairwise comparisons with Bonferroni correction. Error Bars represent ± SEM

(i) *Dopamine:* The NAc showed a mixed impact of surgery and stress that was lateralized to different hemispheres. See Table 3 for significant interaction effects involving stress. In the left NAc, 2 h after the outcome-devaluation test there was a significant and lateralized reduction in dopamine concentration in *Naïve, Sham-injured* and *CCI* rats (*P* < 0.001, Fig. 7). No changes were apparent in dopamine concentration in the right NAc.
(ii) *DOPAC:* 2 h after the outcome-devaluation test, all groups (*Naïve*, *Sham-injured*, *CCI*) showed a significant decrease in DOPAC restricted to the left NAc (*Stress* vs *Stress (*+ *2 h)*) (*P* < 0.001, Fig. 7).
(iii) *HVA:* A significant interaction effect of stress x surgery x side (F_4, 50_ = 2.564, *P* < 0.05) was revealed by the Three-Way Mixed ANOVA. Two hours after outcome-devaluation testing, all groups showed significantly reduced HVA levels in the left NAc (*P* < 0.01, Fig. 7). In addition, *Naïve* rats showed a significant decrease in HVA concentrations in the right NAc (*P* < 0.01) (Fig. 7).

#### Serotonergic Transmission

##### Medial Prefrontal Cortex

**Fig. 8:**
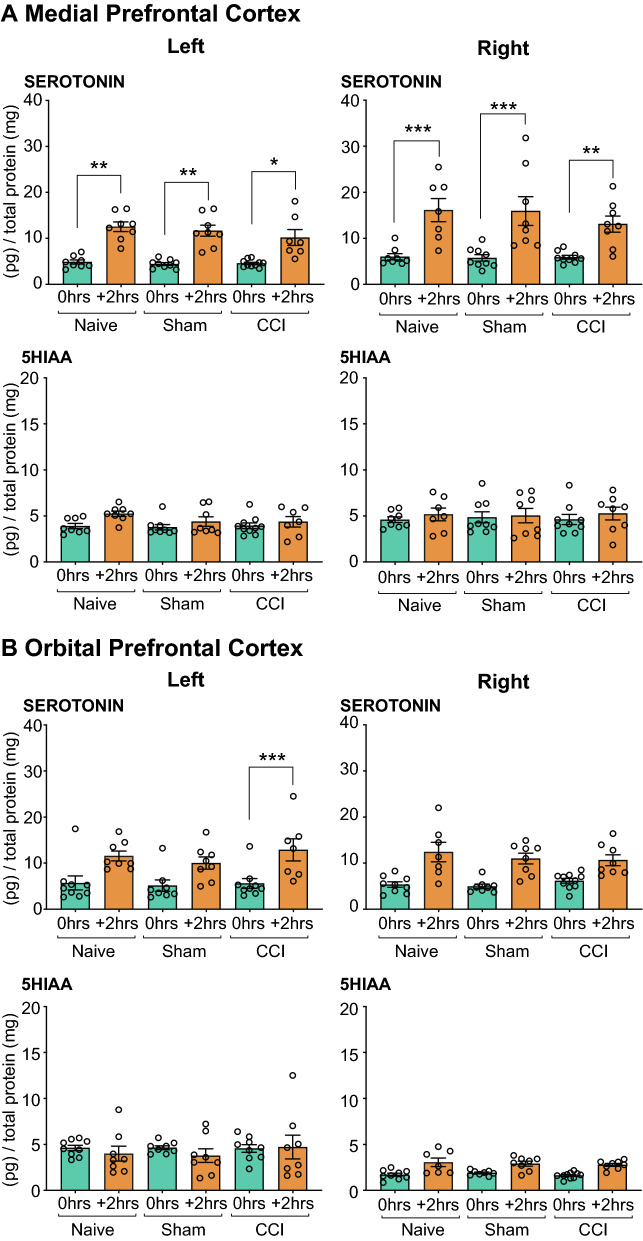
Neurochemical analysis of serotonergic transmission in the **A** Medial Prefrontal Cortex and **B** Orbital Prefrontal Cortex. The serotonin and 5HIAA concentration in the left and right NAc of rats that underwent CCI and acute restraint stress (INJURED + STRESS—*Naïve, Sham, CCI*) are presented. Significant differences are represented by *P < 0.05, **P < 0.01 and ***P < 0.001 when compared + *2 h* are compared to *0 h* groups*.* Three-way ANOVA, with simple effects pairwise comparisons with Bonferroni correction. Error Bars represent ± SEM

(i) *Serotonin:* Significant main effects of stress (F_2, 60_ = 32.948, *P* < 0.001) and side (F_1, 60_ = 23.1, *P* < 0.001) were revealed by the Three-way Mixed ANOVA. Simple pairwise comparisons with Bonferroni correction revealed bilateral increased serotonin levels 2 h after outcome-devaluation testing in *Naïve, Sham-injured* and *CCI* rats (See Table 1 and Fig. 8A).
(ii) *5HIAA:* No significant changes were observed for 5HIAA (see Fig. 8A).

##### Orbital Prefrontal Cortex

(i) *Serotonin:* A statistically significant main effect of stress (F_2, 60_ = 10.157, *P* < 0.001) was revealed by the Three-way Mixed ANOVA. Pairwise comparisons with Bonferroni correction revealed that 2 h after the outcome-devaluation test there was an increase in serotonin concentration in the left oPFC of *CCI* rats only (see Table 1 and Fig. 8B).
(ii) *5HIAA:* No significant changes were observed for 5HIAA (see Fig. 8B).

##### Nucleus Accumbens

**Fig. 9:**
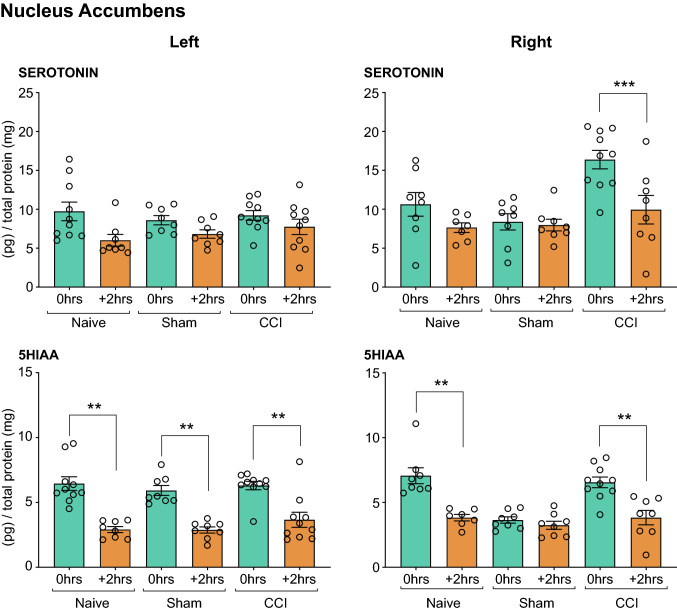
Neurochemical analysis of serotonergic transmission in the Nucleus Accumbens. The serotonin and 5HIAA concentration in the left and right NAc of rats that underwent CCI and acute restraint stress (INJURED + STRESS—*Naïve, Sham, CCI*) are presented. Significant differences are represented by **P < 0.01 and ***P < 0.001 when compared + *2 h* are compared to *0 h* groups*.* Three-way ANOVA, with simple effects pairwise comparisons with Bonferroni correction. Error Bars represent ± SEM

(i) *Serotonin*: The Three-Way Mixed ANOVA revealed statistically significant interaction effects of stress × surgery (F_4, 50_ = 4.567, *P* < 0.01), and significant main effects of stress (F_2, 50_ = 5.317, *P* < 0.01) and side (F_1, 50_ = 4.472, *P* < 0.05). A lateralized effect of acute restraint was apparent in the right NAc of CCI rats immediately after the outcome devaluation test. This effect was abolished 2 h after outcome devaluation (see Table 3 and Fig. 9).
(ii) *5HIAA:* A Three-Way Mixed ANOVA revealed statistically significant main effects of stress (F_2, 50_ = 10.454, *P* < 0.01) and surgery (F_2, 50_ = 4.786, *P* < 0.05). Simple pairwise comparisons revealed a decrease in 5HIAA levels 2 h after outcome devaluation testing in both the right and left NAc, with the sole exception of the right NAc of the *Sham injured* group (*P* < 0.01, Fig. 9).

## Discussion

The aim of this study was to evaluate changes in the neurochemistry of dopaminergic and serotoninergic pathways in rats with neuropathic injuries in response to an acute stressor (INJURED + STRESS) versus rats given neuropathic injury alone (INJURED). Dopamine, serotonin, and their metabolites were measured in the medial prefrontal cortex, the orbital prefrontal cortex, and the nucleus accumbens, brain regions critical for motivated behaviour and decision-making. The key findings reveal that nerve-injury has a selective effect on dopamine activity in the mPFC, and even in rats with nerve-injury (neuropathic pain), acute stress can still trigger specific dopamine responses in the mPFC, and the NAc, and serotonin in the mPFC.

### CCI Decreases Dopamine in the mPFC

Following a CCI in the right sciatic nerve, it was revealed that nerve-injury alone resulted in decreased dopamine in the mPFC. Reduced dopamine signalling is a hallmark of impaired motivation, and is also characteristic of chronic pain states [25]. Human imaging studies in fibromyalgia patients have shown activity in key regions involved in dopaminergic transmission, the ventral tegmental area (VTA) is dramatically reduced or abolished during measurements of hedonic aspects of subjective pain, anticipation of pain relief and pain anticipation [32], and that dopamine release in response to painful stimuli is impaired [33, 34]. These findings are supported by rodent models of chronic neuropathic pain that reveal: reductions in c-Fos expression, a marker of neuronal activation, in the VTA [35]; reduced dopamine release in the mPFC which is associated with increased pain sensitivity [36]; and D2 receptor-mediated suppression of mechanical nociception [37]. The contributions of a hypo-dopaminergic state to chronic pain is also supported by observations of people with Parkinson’s disease for whom pain is a prevalent comorbidity, affecting 40–85% of Parkinsonian patients [38, 39]. Pain in Parkinson’s disease has been reported to occur as early as the prodromal phase, through to the palliative stages of the disease [40]. Further, pain in Parkinson’s can present in multiple forms including, musculoskeletal, central or visceral pain, pain related to motor fluctuations and radicular pain [41]. The presence of painful symptoms related to motor fluctuations such as dystonia (dopamine deficiency) can often be relieved by L-DOPA treatment [41–43]. The pathological dopamine depletion in Parkinson’s disease significantly impacts sensory discriminative aspects of pain detection, with abnormal sensory processing reported during disease development [44].

Prolonged pain is also associated with reductions in the tonic levels of both dopamine and DOPAC in the orbitofrontal cortex of rodents [45]. Deficits in dopaminergic signalling in these cortical regions may contribute significantly to the high incidence of negative affect, impaired cognition, and diminished motivational drive in the chronic pain population. Indeed, the idea of a blunted motivational drive in a chronic pain state is observed in rodent studies. When food is readily available under a fixed ratio operant responding task, there is no difference in effort expended or food consumed between chronic pain or control groups [46, 47]. However, when the amount of effort required to perform the reward-driven action increases (via a progressive ratio schedule), rats with chronic pain are less motivated to obtain food [47].

Further, the prolonged pain triggered by chronic inflammation is associated with increased risky behaviours assessed using a decision-making ‘gambling task’ in rodent subjects [45]. In contrast, in a model of neuropathic pain, rats showed no impairments in goal-directed behaviour despite showing reductions in dopamine in the mPFC (see Mor et al.) [8]. However, here we show that following an acute restraint stress (15 min), nerve-injured rats showed impaired sensitivity to devaluation, despite comparable levels of DA, HVA and DOPAC as sham and naïve groups, which exhibited intact sensitivity to devaluation. In vivo microdialysis experiments following immobilization stress or tail shock stress in rats revealed a similar DA time-course with DA concentrations peaking 15–20 min after the onset of immobilization, with concentrations returning to baseline after 120 min [48, 49]. While in our own experiments the 2 h post-stress interval was chosen mostly for logistical reasons to facilitate tissue collection, additional measures would be informative in revealing the time-course of the stress response in nerve-injured rats. An alternative possibility is that impaired sensitivity to devaluation is due to reduced dopamine binding to both D2/D3 receptors in the face of the acute stress [8, 9].

### Acute Stress Evokes Lateralized Dopamine Increases in the NAc

Immediately following acute restraint, we detected a lateralized increase in dopamine in the left NAc, however this was not related to the CCI as similar increases were seen in the left NAc of both sham and naïve controls. Similarly, no changes in dopamine concentrations were observed following the spared nerve injury model of neuropathic pain [50, 51]. This suggests that dopamine transmission in the NAc following acute stress is stable even in the context of neuropathic injury.

Functional asymmetry in the activity of forebrain dopaminergic pathways arising from the substantia nigra and the VTA has been observed in several experimental contexts [52, 53]. These asymmetries are thought to play a role in a range of affective-motivational behaviours that characterise a number of distinctive behavioural phenotypes. For example, dopamine asymmetry in the NAc, is related to impulsivity, with this region also being responsive to acute stress [54, 55]. The acute stress induced increase in dopamine that we report here may reflect an overall low level of impulsivity in the Sprague–Dawley rats used. What is clear however is that these differences are not related to the outcome-devaluation responses measured.

### Acute Stress Increases Serotonin in the mPFC

Acute restraint increased serotoninergic activity in the mPFC 2 h after the outcome-devaluation test in each of the experimental groups tested. These observations are consistent with in vivo microdialysis studies of serotonin release which show that in response to a psychological stressor, serotonin is released in mPFC as well as the basolateral amygdala [56]. In the mPFC, serotonin concentrations peaked 60 min after stressor exposure and were sustained for a further 20 min, a similar pattern was also observed for the amygdala [56]. This generalized stress-induced increase in serotonin transmission has been shown to play a critical role in the regulation of different behavioural coping styles in rats [57]. For example, rodents with a proactive coping style, exhibit stronger activation of serotonergic neurons in the DRN following exposure to social stress [58]. In mice, De Miguel et al. [59] showed a correlation between increased tryptophan hydroxylase and the expression of active coping behaviours, suggesting a role for serotonin in this specific behavioural response. Experiments in mice have shown that restraint stress increased 5-HT inputs in the mPFC, which resulted in sustained immobility in the forced swim test, a passive coping mechanism [60]. This effect was reversed by serotonin depletion in the mPFC [60].

## Summary

These data suggest that nerve injury has a selective effect on dopamine activity in the mPFC, they also show that despite having nerve injury/neuropathic pain, acute stress can still trigger changes in dopamine in the mPFC, and the NAc; and serotonin in the mPFC.

We suggest that the preservation of the dopaminergic and serotonergic responses to an acute stressor are part of crucial circuitry required to initiate a coping response, and that this circuitry remains active in rats with a neuropathic injury. These data also suggest that the effects of acute stress on outcome-devaluation in nerve-injured rats is independent of the activity of dopaminergic and serotoninergic transmission in the mPFC, oPFC and NAc.

## Data Availability

The datasets generated during and/or analysed during the current study are available on request from the corresponding author.

## Abbreviations

5HIAA: 5-Hydroxyindoleacetic acid
5-HT: Serotonin
CCI: Chronic constriction injury
DA: Dopamine
DOPAC: 3, 4-Dihydroxyphenylacetic acid
EDTA: Ethylenediaminetetraacetic acid
HPLC: High performance liquid chromatography
HVA: Homovanillic acid
mPFC: Medial prefrontal cortex
NAc: Nucleus accumbens
oPFC: Orbital prefrontal cortex
VTA: Ventral tegmental area
